# Stackelberg evolutionary game theory: how to manage evolving systems

**DOI:** 10.1098/rstb.2021.0495

**Published:** 2023-05-08

**Authors:** Alexander Stein, Monica Salvioli, Hasti Garjani, Johan Dubbeldam, Yannick Viossat, Joel S. Brown, Kateřina Staňková

**Affiliations:** ^1^ Centre for Cancer Genomics and Computational Biology, Barts Cancer Institute, Queen Mary University London, London EC1M 5PZ, UK; ^2^ Institute for Health Systems Science, Faculty of Technology, Policy and Management, Delft University of Technology, 2628 BX Delft, The Netherlands; ^3^ Delft Institute of Applied Mathematics, Delft University of Technology, 2628 CD Delft, The Netherlands; ^4^ CEREMADE, CNRS, Université Paris-Dauphine, Université PSL, 75016 Paris, France; ^5^ Department of Integrated Mathematical Oncology, Moffitt Cancer Center, Tampa, FL 33612, USA

**Keywords:** evolutionary game theory, Darwinian dynamics, cancer evolution, fisheries management, optimization, evolutionary rescue

## Abstract

Stackelberg evolutionary game (SEG) theory combines classical and evolutionary game theory to frame interactions between a rational leader and evolving followers. In some of these interactions, the leader wants to preserve the evolving system (e.g. fisheries management), while in others, they try to drive the system to extinction (e.g. pest control). Often the worst strategy for the leader is to adopt a constant aggressive strategy (e.g. overfishing in fisheries management or maximum tolerable dose in cancer treatment). Taking into account the ecological dynamics typically leads to better outcomes for the leader and corresponds to the Nash equilibria in game-theoretic terms. However, the leader’s most profitable strategy is to anticipate and steer the eco-evolutionary dynamics, leading to the Stackelberg equilibrium of the game. We show how our results have the potential to help in fields where humans try to bring an evolutionary system into the desired outcome, such as, among others, fisheries management, pest management and cancer treatment. Finally, we discuss limitations and opportunities for applying SEGs to improve the management of evolving biological systems.

This article is part of the theme issue ‘Half a century of evolutionary games: a synthesis of theory, application and future directions’.

## 1. Introduction

Evolutionary game theory reveals the logic behind adaptations when evolution by natural selection is frequency-dependent [1–3]. Accordingly, an individual’s fitness depends not only on her own trait, but also on the densities of traits in the population. These traits may be simple animal behaviours as suggested by the Prisoner’s Dilemma (PD), Hawk-Dove (HD) and Rock-Scissors–Paper (RSP) games [1,2]. The former two games represent social dilemmas where everyone benefits most when all Cooperate (PD) or all play Dove (HD). Yet, the outcome of natural selection in the PD is Defect (unless one adds iterative plays of the game or non-random interactions), and the HD game generally results in the coexistence of the two strategies. The genius behind Maynard Smith and Price involved their evolutionarily stable strategy (ESS) definition [4]. While Defect in the PD was not a group optimum, it was a strategy that, when common in the population, could not be invaded by any rare alternative strategies. And, while the mixed strategy of the HD game fails the group, it does illustrate what happens when a rare strategy Hawk (or Dove) can invade a population where Dove (or Hawk) are common. Neither Hawk nor Dove is resistant to invasion. The RSP game revealed how the Nash equilibrium (in this case (1/3, 1/3, 1/3)) was not attainable if one included strategy dynamics (a key component of evolving systems). An enduring point of the past circa 50 years of evolutionary game theory is that ESSs, while not always attainable, are more often than not the outcome of evolution by natural selection.

Game-theoretic thinking on natural selection precedes 1973. Lewontin proposed game theory from the perspective of organisms playing against their physical environments [5]. In some ways this is a prescient thought, in that life can be seen as entities that model ('game') the laws of physics and chemistry, and then go on to evolve further adaptations to game conspecifics, competitors, predators and prey. For instance, Fisherian sex ratios where parents invest essentially equal effort into female and male offspring (most notably the 50 : 50 sex ratio) follow from game-theoretic logic [6,7]. Height in trees is an outcome of an evolutionary arms race, and managers in forestry and silviculture consider this when thinning and spacing trees [8–10]. Similarly, evolutionary game theory provides the logic behind the evolution of radical male adornments across many animal species, Batesian mimicry and cannibalism in flour beetles, as well as the evolution of cooperation and mutualisms. All of these topics were noted as frequency-dependent prior to 1973 [11–14].

The outcomes of evolutionary games are driven by natural selection involving both changes to population size (ecological dynamics) and the frequency of heritable traits (evolutionary dynamics). This is not necessarily so for games involving humans. First, humans are rational and can base their decisions on a variety of goals that do not necessarily involve life and death [15,16]. Second, payoffs can involve diverse tangibles and intangibles such as monetary profit, utility, pleasure or aesthetics [17]. Despite differences in the ways that humans and nature plays games, they do come together as bio-economic or bio-sociologic games, in which the actions of humans influence the eco-evolutionary dynamics of pest species, pathogens, commercially or recreationally harvested species, and species of conservation interest. One of the first examples of this dates back to King James I of Scotland. It was brought to his attention that the size of cod seemed smaller than before [18]. This is an early record of how size-selective harvesting of fish causes notable evolutionary changes in size at first reproduction, fecundity, and other life-history traits. Similarly, a scientist from the United States Department of Agriculture noted in the early 1900s how various agricultural pests were evolving resistance to various biocides [19]. Starting in the 1950s, it was recognized how various forms of weed control selected for crop ecotypes of weeds that had adjusted their seedling phenology in response to hand weeding, their seed size in response to sorting techniques, and maturation timing to match harvesting regimes [20,21]. Throw into this antibiotic-resistant strains of bacteria [22] and the evolution of therapy resistance within cancer patients [23], and it is clear that managing evolving species, be they pests, resources, diseases or species of conservation interest, poses unique challenges.

**Figure 1.**
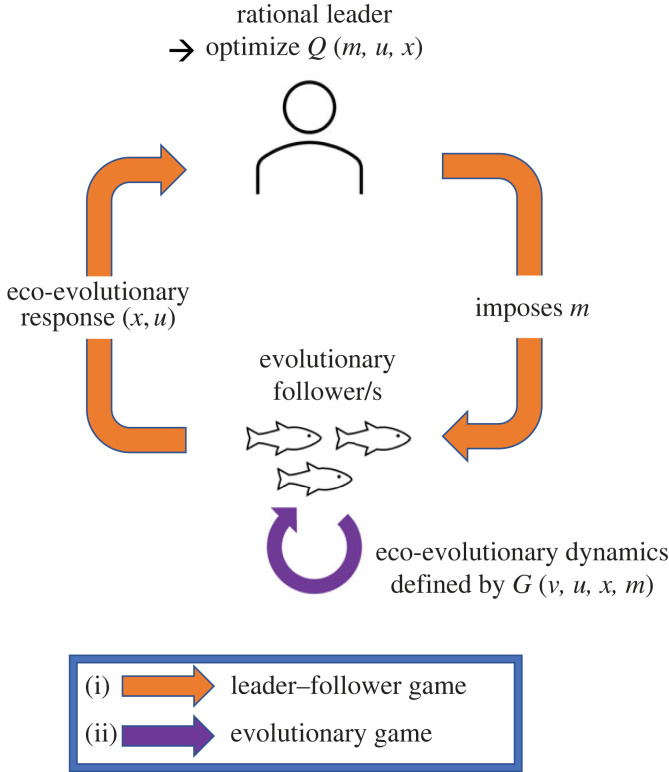
Illustration of the Stackelberg evolutionary game. It combines two types of games: (i) the leader–follower (Stackelberg) game between the rational leader and evolutionary followers, and (ii) the evolutionary game between the followers. The evolutionary game is defined by the fitness-generating function *G*(*v*,*u*,*x*,*m*), which determines the eco-evolutionary dynamics of the followers (§2). In the leader–follower game, the rational leader chooses their strategy *m*, with the goal to optimize their objective function *Q*(*m*,*u*,*x*) (§3). The Stackelberg strategy of the leader anticipates the eco-evolutionary response (*x*, *u*), whereas the Nash strategy anticipates the ecological response *x* only. (Online version in colour.)

Stackelberg evolutionary game (SEG) theory provides a framework for modelling and managing such evolving systems [24]. Its main idea is straightforward. Humans, as managers, stakeholders, or simply as concerned citizens, take actions that directly or indirectly influence the population sizes (ecological dynamics) and evolutionary characteristics (evolutionary dynamics) of species of interest. The species of interest follow the dictates of natural selection and evolutionary game theory. Based on the manager’s actions, there will be changes in the abundance of the species, as well as in their evolutionary traits (figure 1).

Managers and stakeholders can take several approaches. First, they may simply take actions based on the current disposition of the species with respect to the species’ abundance and trait values. In this case, simply weed away without forethought of the eventual consequences. Second, they may consider the ecological consequences of their actions, such as aiming to maintain a sustainable stock of fish while ignoring evolutionary consequences. Third, the manager may anticipate and steer both the ecological and evolutionary consequences of various management strategies for the species of interest. In the first case, the manager is neither ecologically nor evolutionarily enlightened, the second represents an ecologically but not evolutionarily enlightened manager, and the third is both ecologically and evolutionarily enlightened. This third case corresponds to the leader’s Stackelberg strategy in the SEG game. SEGs are characterized by a rational leader and evolutionary followers playing an evolutionary game among themselves [24–26]. The manager has the potential to leverage their advantages because they play first in terms of implementing a set of actions and because they are rational. Based on the manager’s actions, the evolving species evolve to a new ESS (if it exists), according to their eco-evolutionary dynamics. The opportunity here is to promote choices by humans that are both ecologically and evolutionarily enlightened when dealing with the other denizens of our planet. There is already an existing tradition of using evolutionary game theory to solve such bioeconomic games going back to Law and Grey in 1989, when they presented the 'evolutionarily optimal harvest strategy' for managing commercially valuable species, such as fisheries [27]. Our goal here is to offer a general framework for SEGs that can be applied broadly. In what follows, we (i) formalize the notation and framework for SEGs and establish the conditions under which the Stackelberg solution (both ecologically and evolutionarily enlightened) is either the same as or different from and superior to the Nash solution (ecologically but not evolutionarily enlightened), (ii) establish conditions under which the actions of the manager may either decrease or increase the strategies that coexist at the ESS, and (iii) consider applications to fisheries, cancer and pest management. We conclude with a prospectus for what needs to be done regarding the theory and application of SEGs in various domains.

## 2. Formalizing the game among evolutionary followers

Let **x**(*t*) = (*x*_1_(*t*), …, *x*_*n*_(*t*))^*T*^ define population sizes of evolutionary followers with types in Θ={1,…,n} at time *t*. The fitness of a follower of type i∈Θ may depend on both the densities and traits of all followers and the actions of the leader. Consequently, the ecological dynamics of followers of the *i*th type are given by

2.1 $$\frac{dx_{i}(t)}{dt}=x_{i}(t)\cdot H_{i}(U(t),x(t),m(t)).$$

Here, **U**(*t*) = (*u*_*ij*_(*t*)) is the trait matrix at time *t*, where *u*_*ij*_ denotes the value of trait j∈Ψ={1,2,…,p} of a follower of type *i*, and the vector **m**(*t*) = (*m*_1_(*t*), …, *m*_*q*_(*t*))^*T*^ describes the intensities of the *q* possible actions of the leader. Finally, *H*_*i*_(**U**(*t*), **x**(*t*), **m**(*t*)) is the *per capita* growth rate of follower of type *i* at time *t*. It may give rise to both density- and frequency-dependent dynamics, as it depends on **x** explicitly.

The evolutionary dynamics may be described through a *fitness generating function*, or G-function [28]. Such a function describes the fitness *G*(**v**, **U**, **x**, **m**) of a single individual of type **v** = (*v*_1_, …, *v*_*p*_)^*T*^ when the current types, their densities, and the actions of the leader are described by **U**, **x** and **m**, respectively. In particular, replacing *v*_*j*_ in the G-function with *u*_*ij*_ for each j∈Ψ yields the fitness of a follower of type *i*. Thus,

2.2 $$G(v,U,x,m){|}_{v=(u_{i1},\ldots ,u_{ip})}=H_{i}(U,x,m),$$

and equation (2.1) may be rewritten as

2.3 $$\frac{dx_{i}(t)}{dt}=x_{i}(t)\cdot G(v(t),U(t),x(t),m(t)){|}_{v(t)=(u_{i1}(t),\ldots ,u_{ip}(t))}.$$

Followers with a higher *per capita* growth rate will persist in the population. Therefore, the dynamics of trait *j* of a follower of type *i* are given as

2.4 $$\frac{du_{ij}(t)}{dt}=\sigma_{ij}\frac{\partial G(v(t),U(t),x(t),m(t))}{\partial v_{j}(t)}{|}_{v(t)=(u_{i1}(t),\ldots ,u_{ip}(t))}.$$

Here, *σ*_*ij*_ defines the evolutionary speed and is a measure of heritability and additive genetic variance, in line with Fisher’s fundamental theorem of natural selection [29,30]. This speed may be influenced by many other factors, such as mutation rates, population size, population structure and the underlying genetics of inheritance. In *adaptive dynamics*, *σ*_*ij*_ increases linearly with population size, but is stochastic with respect to other variables (canonical equation of adaptive dynamics [31–35]). For the sake of simplicity, when modelling (2.4), it is often assumed that *σ*_*ij*_ is the same constant for all *i* and *j*, while one could easily imagine that *σ*_*ij*_ varies in time and may be a (likely nonlinear) function of *x*_*i*_(*t*), as suggested by adaptive dynamics. In the remainder of this paper, we will not write out the time-dependence explicitly. Thus we shall use **U**, **x** and **m** instead of **U**(*t*), **x**(*t*) and **m**(*t*), respectively. Equations (2.3) and (2.4) constitute the Darwinian dynamics, describing the eco-evolutionary dynamics of evolutionary followers in response to a vector-valued action **m** of the leader.

If the ecological dynamics (2.3) converge to a stable equilibrium x* ≥ 0, we call x* an *ecological equilibrium*. Each combination of followers’ evolutionary traits and leader’s strategies (**U**, **m**) may have an associated vector of stable population sizes x*, with $x_{i}^{*}\geq 0\;\forall i\in \{1,2,\ldots ,n\}$. A generic **U** may correspond to no, one or more values of x*, depending on the G-function. Moreover, even if we assume that the ecological equilibrium exists for any choice of **U** and **m**, only a subset of possible values of **U** and **m** will correspond to positive equilibrium population sizes, where for other values some types of followers will go extinct [36].

Solved together with left-hand side set to zero, equations (2.3) and (2.4) often determine an eco-evolutionary equilibrium solution, a pair composed of followers’ equilibrium population size and trait values, which we will denote by x*(**m**, U*(**m**)) and U*(**m**). It is also possible that none or only one of the dynamics (2.3) and (2.4) is at equilibria. We will discuss that situation as well.

The non-zero equilibrium values of $x_{i}^{*}(m)$ and their associated strategies $\left(u_{i1}^{*}(m),\ldots ,u_{ip}^{*}(m)\right)$ form a ‘coalition’ of strategies. If for a particular choice of **m** these strategies resist invasion by mutant strategies, then they are called ESSs with respect to action **m** [1]. A necessary condition for an ESS is that it maximizes *G* with respect to *v*_*i*_ for those $x_{i}^{*}(m)$ that are positive. This implies that the fitness of a mutant strategy is not larger than the fitness at the ESS [28]. Further stability properties of the ESS can be analysed (e.g. convergence stability or neighbourhood invasion stability (NIS) [37,38]; the extension to the matrix evolutionary traits is straightforward).

## 3. Formalizing the Stackelberg evolutionary game

Here, we will formalize the situation where we include a rational player in the evolutionary game. This additional player (leader) can choose **m** in order to optimize their objective, while the followers’ eco-evolutionary dynamics are described by (2.3) and (2.4). Since the followers are evolutionary players within the structure of a leader–follower (Stackelberg) game [16,39], we call these games SEGs in accordance with recent research on this topic [24,26,40–42]. The leader, as the only rational player in this SEG, is assumed to be able to anticipate and steer the eco-evolutionary responses of the followers defined by (2.3) and (2.4), while followers can only adapt to the actions already taken by the leader.

We will first briefly discuss the situation when neither ecological nor evolutionary equilibria are achieved (yet) (§3(a)) or when the transient dynamics towards equilibria are considered important. Subsequently, we will focus on the most studied case where the eco-evolutionary equilibria are reached and where the strategies of the leader and followers are scalar-valued (§3(b)).

### (a) Stackelberg evolutionary game in transient dynamics

We introduce a rational leader selecting a strategy $m(\cdot )\overset{\mathrm{def}}{=}\;[m(t){]}_{t\in [0,T]}$, where the eco-evolutionary dynamics of the followers are defined through (2.3) and (2.4). Here, $T\in R_{\infty }^{+}=R^{+}\cup \{+\infty \}$ and can also be defined as the first time an ecological and/or evolutionary equilibrium is reached. The objective *Q* of the leader varies with **m**( · ), where $U(\cdot )\overset{\mathrm{def}}{=}\{[U(t){]}_{t\in [0,T]};U(0)=U_{0}\}$ and $x(\cdot )\overset{\mathrm{def}}{=}\{[x(t){]}_{t\in [0,T]};x(0)=x_{0}\}$. In such a situation, the leader’s goal is to find the optimal m*( · ) that maximizes such an objective, i.e. find

3.1 $$m^{*}(\cdot )=\arg\underset{m(\cdot )}{\max}Q(m(\cdot ),U(\cdot ),x(\cdot )),$$

subject to (2.3) and (2.4) and the initial conditions **U**(0) = **U**_0_ and **x**(0) = **x**_0_. The problem defined by (3.1) with respect to (2.3) and (2.4) is an optimal control problem. Thus we could use open-loop, closed-loop or feedback strategies to solve it [16]. However, we will focus on the variant of the problem when an ecological equilibrium of the followers has been reached and when the leader’s objective depends only on traits and population size at that equilibrium.

### (b) Simplified variant of the problem

One can consider variants of the problem from §3(a) where either the ecological dynamics (2.3), the evolutionary dynamics (2.4) or both reach equilibria. The former two cases occur due to time-scale separation of the ecological and evolutionary dynamics [43,44], an assumption considered realistic for many eco-evolutionary dynamic systems. When the eco-evolutionary dynamics are very fast or when the transient dynamics are not that important for the problem at hand, one can assume that the eco-evolutionary equilibrium has been reached, while the objective function *Q* can also depend on the transient dynamics leading to this equilibrium. In such a case, *T* may be defined as the first time when the eco-evolutionary equilibrium of (2.3) and (2.4) is reached.

In the next section of this work (§4), we will analyse the simplest possible version of the problem (3.1) with respect to (2.3) and (2.4). We will assume the following:

A1. As opposed to the vector- or matrix-valued dynamics (2.3) and (2.4), the evolutionary and ecological traits of the followers are scalar, and their population is monomorphic, with the eco-evolutionary dynamics defined as
  3.2 $$\frac{dx}{dt}=x\cdot G(v,u,x,m){|}_{v=u}$$
  and
  3.3 $$\frac{du}{dt}=\sigma \frac{\partial G(v,u,x,m)}{\partial v}{|}_{v=u}.$$
A2. The leader searches constant *m* maximizing *Q*(*m*, *u*, *x*).
A3. The objective of the leader *Q*(*m*, *u*, *x*) is differentiable and defined at the ecological equilibrium of the system x*(*m*, *u*).

Given A1–A3, we will consider different assumptions regarding the leader’s knowledge of the eco-evolutionary equilibria of (3.2)–(3.3) when optimizing their objective, which will lead to different outcomes of this leader’s optimization.

Most of the results we will present in the next section can be extended to the more generic cases when these assumptions are relaxed. When discussing different applications of SEGs (§5), we will consider both the simplest and more generic forms of the game, with followers’ eco-evolutionary dynamics defined by (2.3) and (2.4).

## 4. Properties of Stackelberg evolutionary games at ecological equilibrium x*(*m*, *u*)

### (a) Evolutionary response of followers at x*(*m*, *u*)

Let us assume that for a fixed *m* and *u*, the population reaches an equilibrium x* = x*(*m*, *u*) (ecological equilibrium) where x*(*m*, *u*) is defined by *G*(*v*, *u*, x*(*m*, *u*), *m*)|_*v*=*u*_ = 0 when x*(*m*, *u*) is positive. The ESS strategy u*(*m*) of the followers in response to the leader’s strategy *m* maximizes *G* as follows:

4.1 $$u^{*}(m)=\arg\underset{v}{\max}G(v,u^{*}(m),x^{*}(m,u^{*}(m)),m).$$

The expression u*(*m*) represents the best response of the followers, in accordance with the dynamic game theory literature [16]. While we assume that the followers’ evolutionary strategy will reach (4.1) and that the system is at ecological equilibrium x*(*m*, *u*), the leader may or may not consider these pieces of information. That brings us to the possible strategies of the leader.

### (b) Leader’s possible strategies at x*(*m*, *u*)

The leader can be naive, ecologically enlightened or evolutionary enlightened. There are two possible interpretations of a naive strategy by the leader: (i) either the leader maximizes their objective with respect to *m*, while not taking eco-evolutionary dynamics into account, or (ii) the leader plays an *a priori* constant action, which in practice often corresponds to the maximum possible action, in the belief that this is the best possible action to play, not optimizing anything. In this paper, we will assume that these two actions coincide. If the leader takes the ecological dynamics into account, they will maximize *Q*(*m*, *u*, x*(*m*, *u*)). As explained in the electronic supplementary material, this eventually leads us to the Nash equilibrium (formally defined below). Finally, if the leader additionally takes the followers’ evolutionary dynamics (4.1) into account they will maximize *Q*(*m*, u*(*m*), x*(*m*, u*(*m*))), leading to the Stackelberg equilibrium.

The three possible strategies of the leader can be formalized as follows:

**Naive strategy:** The leader plays a constant and aggressive strategy *m* = *m*_max_, ignoring followers’ ecological and evolutionary dynamics.

**Ecologically enlightened strategy corresponding to the Nash strategy:** The best response of the manager to the followers is

4.2 $$m^{*}(u)=\arg\underset{m}{\max}Q(m,u,x^{*}(m,u)),$$

the followers respond by their ESS u*(*m*) given by (4.1). A Nash equilibrium (*m*^*N*^, *u*^*N*^) is defined as a pair of strategies that correspond to best responses of the leader and followers to each other, which is given by an intersection of the curves *m* = m*(*u*) and *u* = u*(*m*). At Nash equilibrium, no player can improve their outcome by unilaterally changing their strategy.

**Evolutionarily enlightened leader’s strategy corresponding to the Stackelberg strategy:** With this strategy, the leader anticipates u*(*m*) and x*(*m*, u*(*m*)) and, therefore, can include them both into their objective *Q* before maximizing it with respect to their action *m*. The leader’s Stackelberg strategy is thus

4.3 $$m^{S}=\arg\underset{m}{\max}Q(m,u^{*}(m),x^{*}(m,u^{*}(m))).$$

In all three cases, we assume that the followers are bound to their ESS strategy u*(*m*). At the equilibrium, the evolutionarily enlightened leader can never perform worse than an ecologically enlightened leader. Similarly, the ecologically enlightened leader typically performs better than the naive leader. Of interest is when the Nash and Stackelberg strategies of the leader coincide. The following theorem elucidates that.

**Theorem 4.1 (Equivalence of Nash and Stackelberg equilibria).**

*If a leader’s Nash and Stackelberg strategies are characterized by first-order optimality conditions, then they coincide in the following cases:*

(a) *If* du*(*m*)/d*m* = 0.
(b) *If* (∂*Q*/∂*u*)(*m*, u*(*m*), x*(*m*, u*(*m*)) = 0 *and, moreover*,
  $$\frac{\partial Q}{\partial x}(m,u^{*}(m),x^{*}(m,u^{*}(m))=0$$
  *or*
  $$\frac{\partial x^{*}}{\partial u}(m,u^{*}(m))=0.$$

**Proof.**

The first-order optimality condition for the leader’s strategy to be a Nash strategy is

4.4 $$\frac{\partial Q}{\partial m}+\frac{\partial Q}{\partial x}\frac{\partial x^{*}}{\partial m}(m,u^{*}(m))=0,$$

while the first-order optimality condition for the leader’s strategy to be a Stackelberg strategy is

4.5 $$\begin{array}{rl} \frac{\partial Q}{\partial m} & +\frac{\partial Q}{\partial x}\frac{\partial x^{*}}{\partial m}(m,u^{*}(m))\\ & +\frac{du^{*}(m)}{dm}\left(\frac{\partial Q}{\partial u}+\frac{\partial Q}{\partial x}\frac{\partial x^{*}}{\partial u}(m,u^{*}(m))\right)=0, \end{array}$$

where in both (4.4) and (4.5), all partial derivatives of *Q* are evaluated at (*m*, u*(*m*), x*(*m*, u*(*m*)). It follows that if conditions (a) or (b) are satisfied, (4.4) and (4.5) coincide. ▪

**Remark 4.2.**

Case (a) would arise if the leader’s strategy does not affect the evolution of the trait (at least for relevant values of *m*). There is then no loss in not taking this evolution into account, so it suffices for the leader to be ecologically enlightened.

Case (b) would arise if the following conditions are satisfied: first, the objective function is independent of *u* (the leader only cares about the population size and not the trait of the species), so that ∂*Q*/∂*u* = 0. Second, competition among followers is purely density-dependent, so that $G(v,u,x,m)=\hat{G}(v,x,m)$, with $\hat{G}$ non-increasing in *x*. As shown below, this implies that at the eco-evolutionary equilibrium, the partial derivative of *x* with respect to *u* cancels: (∂x*/∂*u*)(*m*, u*(*m*))) = 0, thus conditions (b) are met.

To see why this derivative cancels, note that at the eco-evolutionary equilibrium (u*(*m*), x*(*m*, u*(*m*)), the fitness of a mutant trait is no larger than the fitness of the resident. Moreover, at any (positive) ecological equilibrium x*(*m*, *u*), the fitness of trait *u* is zero. Therefore, for any trait *v*,

4.6 $$\begin{array}{rl} G(v,u^{*}(m),x^{*}(m,u^{*}(m)),m) & \leq G(u^{*}(m),u^{*}(m),x^{*}(m,u^{*}(m)),m)\\ & =0=G(v,v,x^{*}(m,v),m). \end{array}$$

Since $G(v,u,x,m)=\hat{G}(v,x,m)$, it follows that $\hat{G}(v,x^{*}(m,$ $u^{*}(m)),m)\leq \hat{G}(v,x^{*}(m,v),m)$. Since $\hat{G}$ is non-increasing in *x*, this implies that x*(*m*, u*(*m*)) ≥ x*(*m*, *v*). That is, u*(*m*) maximizes the population size x*(*m*, *u*). Therefore, (∂x*/∂*u*)(*m*, u*(*m*)) = 0, hence the result.

## 5. Applications of Stackelberg evolutionary games

SEG theory can be used in any situation when a rational party (leader) wants to save, contain or eliminate a biological system responding to the leader’s action according to the principles of natural selection. Here, we provide examples of research that has framed such interactions through SEGs and highlight opportunities for further applications of game theory to these domains.

### (a) Fisheries management

In [24], an SEG between a fisheries manager as a rational leader and a fish stock as evolutionary followers was considered. In this model, the leader selects the harvesting rate *m* and the fish respond by evolving their body size at maturation *u* to maximize their fitness. The leader aims at maximizing their net profit *Q*(*m*, *u*, *x*), which is a function of the strategies of the players and the population size of the fish *x*. Following [45], the profit is given by the difference between the value of harvested fish biomass and the cost of fishing, with the harvest coming from two sources: harvesting of adult fish and harvesting of juvenile fish that are larger than the net size, which can be considered as a second decision variable of the manager.

**Figure 2.**
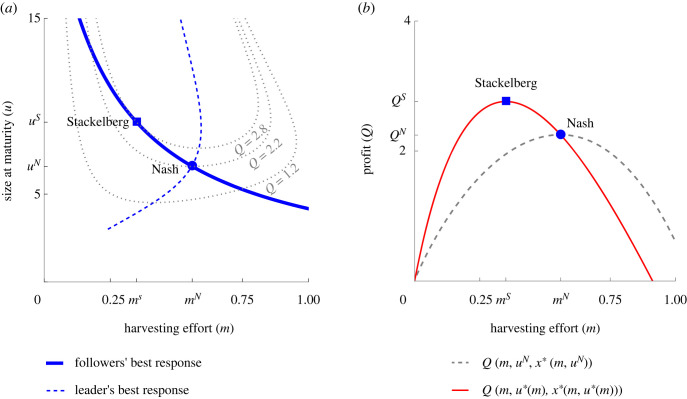
(*a*) The best response curve (ESS) of the fish (bold solid line) and the best response curve of the fisheries manager (bold dashed line). The Nash equilibrium lies at the intersection of the fish’s ESS curve and the best response curve of the manager, while the Stackelberg equilibrium lies on the ESS curve of the fish but not on the best response curve of the manager. This is because the latter one is obtained by maximizing the profit over the best response of the followers. With the Stackelberg approach, the manager adopts a lower harvesting effort, which leads to bigger fish size. (*b*) The effect of the harvesting effort on the profit for the ecologically enlightened strategy (Nash) and evolutionarily enlightened strategy (Stackelberg). The ecologically enlightened manager considers the size of fish at maturation as fixed (*u* = *u*^*N*^) and selects the harvesting rate that maximizes the profit with this in mind (grey dotted curve). The evolutionarily enlightened manager assumes that the size of fish at maturation is the ESS (u*(*m*)) and selects a harvesting rate that maximizes the profit accordingly (red curve). The evolutionarily enlightened approach leads to higher profits with a lower harvesting rate than the ecologically enlightened one. Adapted from [24]. (Online version in colour.)

Figure 2 compares two management strategies of the fisheries manager: ecologically enlightened (Nash) and evolutionarily enlightened (Stackelberg), showing their impact on the fish size and on the profit of the manager. The Nash equilibrium is reached where the best response curve (ESS) of the fish intersects with that of the manager (figure 2*a*). At this point, the fish are evolutionarily stable (as no individual can increase its fitness by unilaterally changing its size) and ecologically stable (as their expected *per capita* growth rate is 0 at x*). For the manager, this is a no regret strategy: given the size of the fish, the manager has no incentive to change the harvesting rate *m*^*N*^. Conversely, the Stackelberg equilibrium is not a point on the manager’s best response curve, but a point on the fish ESS curve where profit is maximized (figure 2*a*).

In practice, the difference between the two management strategies lies in the assumptions. The ecologically enlightened manager recognizes the effects of harvesting on the population size of the fish, but sees the adult size of the fish as fixed, and therefore does not take evolution into account. In order to determine the optimal harvesting rate *m*^*N*^, this manager considers the effect of *m* and x*, but maximizes the profit function *Q* holding *u* constant (figure 2*b*). Conversely, the evolutionarily enlightened manager anticipates that the fish will evolve in response to harvesting, incorporates both the ecological and the evolutionary consequences (x*(*m*, u*(*m*)) and u*(*m*)) of harvesting into the profit function *Q*, and selects the harvesting rate *m*^*S*^ that maximizes the profit with this in mind (figure 2*b*). The profit curve for this management strategy intersects the profit curve for the ecologically enlightened management strategy at its maximum (Nash outcome), meaning that the Nash outcome is achievable for the Stackelberg manager but not *vice versa*. Overall, with the Nash approach, the manager tends to adopt a high harvesting rate that eventually leads to smaller fish (figure 2*a*). With the Stackelberg approach, the manager scales back the harvesting rate, which leads to bigger fish size and higher profit.

In [24], each choice of the fisheries manager (*m*) corresponded to a unique ESS (u*(*m*)) of the fish. However, the manager’s actions may change not only the exact value of the follower’s ESS but also the number of strategies comprising the ESS, possibly due to a speciation event (see also [46]). To see how a biological system with multiple ESSs can occur and what it means for the leader’s best strategies, let us consider another example, with eco-evolutionary dynamics of the fish in the form of (3.2) and (3.3). The eco-evolutionary dynamics are defined through G-function

5.1 $$G(v,u,x,m)=r\left(1-\frac{x}{K(v)}\right)-H(v,m),$$

where $K(v)=K_{\max}\;e^{-v^{2}/\sigma_{K}^{2}}$ is the carrying capacity. Here, $H(v,m)=me^{-v^{2}/\sigma_{H}^{2}}$ defines the harvesting rate with harvesting effort *m* and *u* is the fish’s evolutionary trait related to their catchability (parameters *σ_K_* and *σ_H_* tune the shape of functions *K* and *H*). If there is no harvesting (*m* = 0), the population follows a logistic growth and eventually adopts trait *u* = 0 to maximize the carrying capacity. Increasing the harvesting effort (*m* > 0) leads to reduction of the growth rate by the harvesting rate *H*(*v*, *m*). The manager’s profit is defined as

5.2 Q(m,u,x)=H(u,m)x−cm,

where the first term defines the harvested amount of fish and the second term defines the cost of harvesting, with *c* > 0. In this model, we assume there is some intermediate strategy that maximizes the fishes' access to resources, and we normalize this to *u* = 0. This strategy might be habitat choice, seasonal movements, other foraging strategies, or morphology, all important for determining fish abundance. Hence *u* = 0 maximizes carrying capacity. Furthermore, we assume that investments in fishing gear, fishing boats and fishing regulations have been adjusted and fixed over time to be maximally efficient at catching fish with trait *u* = 0. Therefore, both the carrying capacity of the fish and their catchability are maximized when *u* = 0, and both decline as the fishes' strategy deviates either above or below this value.

**Figure 3.**
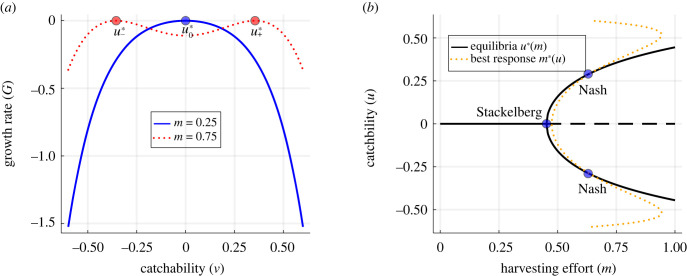
Bifurcation of an ESS of the fish with respect to harvesting rate. (*a*) G-function at the eco-evolutionary equilibrium for fixed *m*. At *m* = 0.25 < *m*_*c*_, there is one ESS ($u_{0}^{*}$), while for *m* = 0.75 > *m*_*c*_, there are two ESS ($u_{\pm }^{*}$). (*b*) A pitchfork bifurcation in ESS at *m*_*c*_ ≈ 0.45. The leader’s best response curve is depicted in dotted orange. Parameter values: *r* = 1.0, *K*_max_ = 10 000, *σ*_*K*_ = 0.55, *σ*_*H*_ = 0.50 and *c* = 500. (Online version in colour.)

The fish are under stabilizing selection to maximize their carrying capacity in the absence of harvesting and under disruptive selection to avoid harvesting. For small harvesting efforts *m*, we find a unique ESS $u_{0}^{*}=0$ that maximizes *G*(*v*, *u*, *x*, *m*) (see figure 3*a*, blue line). As *m* increases, the disruptive selection can turn the convergent stable maximum $u_{0}^{*}=0$ into a convergent stable minimum, thus creating a bifurcation in the ESS into an evolutionarily stable set (ESSet) with two coexisting strategies $u_{+}^{*}$ and $u_{-}^{*}$ (see figure 3*a*, red dotted line). The corresponding bifurcation diagram is depicted in figure 3*b*. With the given parameters, the harvesting effort at which the bifurcation occurs is *m*_*c*_ = 0.45. We see that for *m* < 0.45 the ESS is given by $u_{0}^{*}=0$. At *m* = 0.45, the ESS splits into two branches $u_{+}^{*}$ and $u_{-}^{*}$ that further diverge from each other as *m* increases.

Next, we analyse the Nash and Stackelberg strategies of the game. We calculate the Nash equilibrium (*m*^*N*^, *u*^*N*^) and the Stackelberg equilibrium (*m*^*S*^, *u*^*S*^). First, we consider the case where m*(0) ≤ *m*_*c*_. In this case, the Nash strategy *m*^*N*^ falls below the critical harvesting effort *m*_*c*_ and the corresponding trait value is *u*^*N*^ = 0. We find that the Stackelberg strategy coincides with the Nash strategy, which goes in agreement with theorem 4.1 as du*(*m*)/d*m* = 0 when *m* < *m*_*c*_. When m*(0) > *m*_*c*_, the best response m*(*u*) intersects with both $u_{+}^{*}$ and $u_{-}^{*}$ such that *m*^*N*^ > *m*_*c*_ and *u*^*N*^ ≠ 0. This leads to a change in trait *u* and potentially speciation of the fish. In contrast to the previous case, the Stackelberg equilibrium does not coincide anymore with the Nash equilibrium. Rather, the Stackelberg strategy *m*^*S*^ is equal to the critical harvesting effort *m*_*c*_, thus harvesting as much as possible while keeping the carrying capacity maximized with *u*^*S*^ = 0, as illustrated in figure 3*b*.

Mathematical details and calculations can be found in the electronic supplementary material, and Mathematica code is provided online (see Data accessibility).

### (b) Cancer treatment

**Figure 4.**
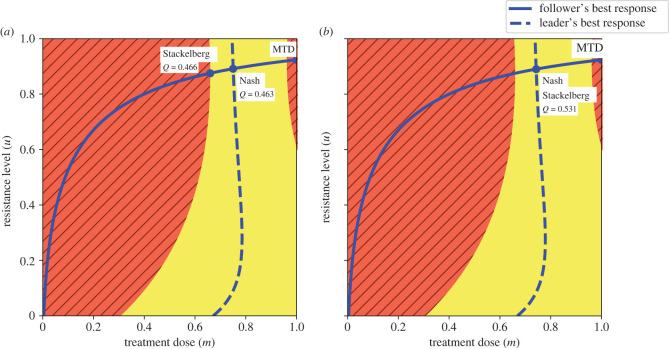
The outcomes of the maximum tolerable dose (MTD), ecologically enlightened (Nash) strategy and evolutionarily enlightened (Stackelberg) strategy of the physician, when playing an SEG against cancer: the yellow and red/cross-hatched areas represent tumour stabilization (0 < x* ≤ *δK*) and progression (x* > *δK*) regions, respectively [42]. (*a*) The Nash and Stackelberg outcomes differ when *Q* defined by (5.3) is an explicit function of *u*. (*b*) The Nash and Stackelberg outcomes coincide when *c*_2_ = 0. Parameterization: *δ* = 0.7, *r*_max_ = 0.45, *g* = 0.8, *K* = 10 000, *d* = 0.01, *k* = 2, *b* = 10, *α*_*SS*_ = *α*_*RR*_ = 1, *α*_*SR*_ = 0.1, *α*_*RS*_ = 0.9, *σ*_*S*_ = 0, *σ*_*R*_ = 1, *Q_max_* = 1; (*a*) *c*_1_ = 0.54, *c*_2_ = 0.21, *c*_3_ = 0.25, (*b*) *c*_1_ = 0.68, *c*_2_ = 0, *c*_3_ = 0.32. (Online version in colour.)

Applications of game theory to understanding cancer and improving treatment have been summarized in a review paper [41] and in multiple publications on this topic [47–50]. To describe how the SEG theory can be useful in improving cancer treatment, let us consider an SEG of cancer treatment between a physician and a polymorphic population of cancer cells consisting of resistant and sensitive cells. This game is based on a model presented by Pressley *et al.* [51]. We extend it by including competition among cancer cells [42]. This inclusion likely makes the model more realistic [52] and the eco-evolutionary dynamics more stable [53]. The sensitive and resistant cells have population *x*_*S*_ and *x*_*R*_, respectively, and resistance traits *u*_*S*_ and *u*_*R*_, respectively, while *m* represents the drug dosage of a single drug. Here, *m* = 0 and *m* = 1 correspond to no dose and the maximum tolerable dose (MTD), respectively. As in [51], the sensitive cancer cells remain drug-sensitive (*u*_*S*_ is always 0), while the resistant subpopulation has a resistance trait that evolves in response to the dose *m* of the drug applied by the physician. The eco-evolutionary dynamics of the cancer cells for each cancer subpopulation *i* ∈ {*R*, *S*} is a simplified case of (2.3) and (2.4) where we have a vector **u** instead of the matrix **U**. In this model, *σ*_*i*_ is the evolutionary speed of the populations *i* ∈ {*R*, *S*}, with *σ*_*S*_ = 0. The eco-evolutionary dynamics are defined using G-function

$$G(v,u,x,m)=r(v)\left(1-\frac{\sum_{\;j\in \{R,S\}}\alpha_{ij}x_{j}}{K}\right)-d-\frac{m}{k+bv},$$

where *r*(*v*) = *r*_max_e^−*g* *v*^ is the growth rate carrying a cost of resistance regulated by *g*, *α*_*ij*_ defines the competitive effect of type *j* on type *i*, *K* is the carrying capacity and *d* the natural death rate. Parameter *k* defines the innate resistance that may be present before drug exposure and *b* the benefit of the evolved resistance trait in reducing therapy efficacy [51]. Our model assumes that depending on the population size at the equilibrium ($x^{*}=x_{S}^{*}(m,u_{S})+x_{R}^{*}(m,u_{R})$), there are three possible outcomes: (i) extinction (x* ≤ 0) where cancer is cured, (ii) progression (x* larger than a certain fraction of the carrying capacity *δK*) where the disease progresses and (iii) stabilization (0 < x* ≤ *δK*) where the cancer can be stabilized as a chronic disease with no or little side-effects related to the tumour burden. The evolutionary response is $u_{S}^{*}=0$ for the sensitive cancer population and is calculated through (4.1) for the resistant cancer population. This is also illustrated in figure 4.

The physician optimizes the constant treatment dose *m* ∈ [0, 1], in order to maximize patient’s quality of life

5.3 $$Q(m,u_{R},x^{*})=Q^{\max}-c_{1}{\left(\frac{x^{*}}{K}\right)}^{2}-c_{2}u_{R}^{2}-c_{3}m^{2},$$

where *Q*^max^ is the maximum quality of life and weights *c*_1_, *c*_2_ and *c*_3_ indicate the extent by which quality of life decreases with the tumour burden, rate of resistance and drug toxicity, respectively. Figure 4 demonstrates how MTD, commonly applied as the standard of care, leads to cancer progression. When disease stabilization is feasible, we compare the physician’s ecologically enlightened and evolutionarily enlightened treatment strategies. The physician’s best response is calculated through (4.2). Figure 4 illustrates that for the considered parametrization, both Nash and Stackelberg solutions stabilize the tumour burden and succeed over MTD, which leads to disease progression. As illustrated in figure 4*a*, the evolutionarily enlightened (Stackelberg) strategy corresponds to both a lower treatment dose/toxicity and a lower treatment-induced resistance than the ecologically enlightened (Nash) one. Furthermore, the Stackelberg strategy leads to the best result in terms of patient quality of life, followed by the Nash strategy, while MTD leads to progression. Figure 4*b* demonstrates a situation when the quality of life function does not include treatment resistance, condition (b) of theorem 4.1 is satisfied, and therefore the Nash and Stackelberg equilibria coincide.

Other examples in cancer treatment where SEG theory could be useful exist. A special case of cancer is transmissible cancer, i.e. cancer that can be transmitted from one individual to another one. While such cancers are currently rare, it is possible that they were much more common during the evolutionary history of life on earth and that over time, the species evolved prevention and suppression mechanisms [54–57]. Tasmanian devils’ facial tumours and clam leukaemia represent examples of such cancers. While it is possible to model cancer spread within one host through our equations (2.3) and (2.4), to frame transmissible cancers within the SEG framework, one needs to include the possibility of cancer transmission from host to host. Spatially implicit or explicit modelling may need to be included in (2.3) and (2.4) for this purpose.

### (c) Pest management

For over 100 years, it has been recognized that insect pests evolve pesticide resistance. More recently, managers have advocated for resistance management plans, including a restrained use of pesticides, crop rotation, a strategic timing of multiple pesticides and pesticide-free sanctuaries [58–60]. SEG theory provides a conceptual framework for targeting pests’ resistance strategies in response to control strategies of the pest manager and the subsequent selection for the best control strategies. The SEG theory can help to replace the currently used ecologically enlightened application of pesticides with evolutionarily enlightened strategies, which will lead to a higher chance for pest containment [61]. Future research can focus on including vector-valued strategies of the pest manager. Those correspond to multiple pesticides and other possible strategies, while the trait strategies should be matrix-valued as in (2.4) if multiple pests are considered.

### (d) Other applications

SEGs frame situations where one tries to control evolving biological systems. There are plenty of examples from the literature where an SEG philosophy is already considered, even if the underlying SEG dynamics are not framed in game-theoretic (or any other mathematical) terms and the leader’s strategies are not explicitly optimized. For instance, the strategy of stabilization of an incurable disease has been successfully applied when treating human immunodeficiency virus infection [62–64] and diabetes [65,66]. It has been recognized that when biomedical interventions fail in curing a disease, it may be better to aim for its control/stabilization. Iwasa *et al.* [67] quantified the probability of disease escape from biomedical interventions, such as vaccines or therapy. Here, a more formal usage of SEGs could help to find better strategies targeting the disease.

Conservation biology, where the goal is the survival of species in deteriorating habitats, is another field where SEGs could be used to achieve better outcomes. Klausmeier *et al.* [68] formulated eco-evolutionary dynamics similar to (2.3) and (2.4) and its extension to the SEG framework is straightforward. Optimizing available conservation strategies by defining a proper objective for the human as a leader in this game is a natural next step.

The idea that the conservation of species is a problem complementary to eradicating species is not novel. The phenomenon of a species’ adaptation to its environment where this adaptation leads to its survival (which is not always wished for) is referred to as evolutionary rescue [69].

SEGs have the potential to be extended and applied in situations leading to global threats to human health, such as preventing antibiotic resistance or containing viral diseases, such as COVID-19 [70,71]. When targeting antibiotic resistance, one could consider the objective function defining a treatment success with the given drug while avoiding the treatment-induced resistance. The human influence on the evolution of antibiotic resistance has been studied in [70] and has the potential to be solved through the SEG theory. The ongoing threat of COVID-19 demonstrates the importance of understanding the ecology and evolution of infectious diseases and subsequent design of appropriate containment strategies [72]. Humans influence the eco-evolutionary dynamics of infectious diseases in multiple ways. Rogalski *et al.* [73] has proposed that framing the problem as an SEG may lead to better therapeutic and non-therapeutic interventions and overall higher quality and quantity of life.

## 6. Discussion

Starting in 1973, the subdiscipline of evolutionary game theory began to expand rapidly [3,4]. It initially did so quite independently of the larger field of ‘classical’ game theory that has been applied to economics, sociology, military sciences, engineering, diplomacy, political sciences and more [16]. This larger domain of game theory beyond evolutionary game theory (EGT) included diverse solution concepts and ways to frame the strategy sets, payoffs and objectives (such as utility, profit, well-being, various societal metrics, tactical or strategic level military or conflict outcomes). Initially, EGT centred around the ESS as a likely outcome of evolution by natural selection. It is well established that an ESS needs to be a Nash equilibrium that is additionally uninvadable [1,74]. EGT has expanded solution concepts to include convergence stability, neighbourhood invader strategy and mutual invasibility [37,38,75]. All developed concepts derive from the ecological and evolutionary dynamics that drive changes in strategy frequencies, in contrast to the rational choice of the classical games [28,76]. Here, we draw on the Nash and Stackelberg solutions from classical game theory and the associated eco-evolutionary dynamics and ESS to formalize games between a rational leader and evolving followers termed SEG Theory in earlier work [24,40]. The need for such formalization begins with the long-term recognition that human management strategies impact not only the distribution and abundance of species (ecological dynamics) but also the evolutionary trajectories of pest species, harvested species, species of conservation interest and diseases. The need is even greater as we see rapid evolution continuing to occur with additional responses to urbanization (evolution of urban eco-types) and climate change (acclimation followed by adaptation of many affected species). Starting with Law and Grey [27], EGT has been applied to these human-nature games in a manner we would term SEGs. Humans can act as rational players in line with classical game theory, and nature responds to human actions in accord with EGT. Prior SEG-like models have been applied to fisheries management, pesticide management, antibiotic resistance, and increasingly in managing therapy resistance when treating cancer [24,26,61]. Here, we begin a unified modelling framework for considering SEGs that can apply to prior, present and future applications. In an SEG, a manager or stakeholder selects an action that aims to maximize their objective in terms of benefiting from a valued species or controlling an undesirable one.

This leads to three management strategies: naive, ecologically enlightened and evolutionarily enlightened. The naive manager takes the current population size and trait value of the species as fixed and bases their choice on whatever are their current values. This tends to lead to overfishing, maximum tolerable dosing in cancer, and generally an extreme strategy by the manager, particularly when the objective is monotonic with respect to the manager’s action. The ecologically enlightened manager considers in advance the consequences of their actions for the species’ population size and adjusts their strategy accordingly. Such a strategy forms the basis of most maximum sustainable harvest style strategies for harvesting species or the individual-level desire to use antibiotics or pesticides. This strategy leads to a Nash equilibrium between the manager’s strategy and the species’ ESS. The evolutionarily enlightened manager considers the eco-evolutionary consequences of their strategy for the species’ ESS. This leads to a Stackelberg solution.

The three management strategies will be the same if the manager’s action does not affect on the species’ strategy or population size—unlikely in virtually all real-life scenarios. Furthermore, in most cases, the naive manager will lead to the most extreme management choices (this can be seen in certain fisheries management strategies that include overharvesting followed by no harvesting at all upon the fisheries’ collapse). The Nash and Stackelberg solutions of the ecologically and evolutionarily enlightened managers, respectively, will be the same if (i) the manager’s actions have no evolutionary effects on the species, or (ii) the species trait has no effect on the manager’s objective, and the species population size does not influence the manager’s objective or the effect of the species strategy on its population size cancels at the ESS. Otherwise, the Stackelberg solution deviates from the Nash. This is likely the case in most realistic scenarios, as the manager’s actions do influence the traits of the species, the species’ trait likely influences its population size, and the manager’s objective likely includes caring about the species trait and population size.

It seems in the models to date that the Stackelberg solution results in a more moderate management strategy than the Nash in terms of harvesting effort, pesticide application or drug therapy (be it in the context of cancer or infectious diseases). Increasing knowledge of the system is required in going from naive to ecologically enlightened to evolutionarily enlightened management. In order to anticipate and steer the eco-evolutionary response of a biological system, we need to improve our ability to estimate population size and composition prior to intervention. In order to estimate and optimize the model parameters, a continuous surveillance is required. We do not yet have sufficient technology for identifying, quantifying and monitoring the evolving strategy distribution in heterogeneous populations. This presents one limit to achieving a Stackelberg solution—although pest management and especially cancer therapies are moving in that direction, with the advent of liquid biopsies, radiomics, organoids and xenografts [77–80].

Another impediment to implementing a Stackelberg solution includes disagreement and lack of knowledge regarding the speed of evolution, and even what trait might evolve. For instance, cancer cells may have more than one resistance mechanism for a given cancer and therapy. Which mechanism will actually evolve in response to therapy, and will it be the same for each patient? This suggests that management strategies might begin with an ecologically enlightened approach as a probe to see in what direction and how quickly the evolutionary traits of the species evolve. Other issues arise when the trait may be qualitative rather than quantitative. For cancer, qualitative resistance might be a result of genetic mutations [81,82]. The management of a pre-existing mutant population was, for example, studied in [83]. However, additional resistance and driver mutations can emerge during therapy, leading to more aggressive cancers. The risk of acquiring additional driver mutations is higher if the tumour burden is high. This needs to be considered for future studies and could be taken into account by implementing a penalty term for high tumour burden in the objective function.

As shown in §5a for fisheries management, the way to the Nash and Stackelberg solutions is not always straightforward, as the followers may speciate into two or more types with distinct strategies. While the discussion about speciation and its impact on finding the Nash and Stackelberg equilibria has just started, it becomes natural to look in the direction of adaptive dynamics as a natural extension to our work [75,84]. For instance, a fitness minimum may represent an evolutionary branching point of adaptive dynamics [32,85,86], leading to different eco-evolutionary responses in the followers from those that we defined.

Our presentation of SEG theory is just a beginning and invites more expansive models, theorems and applications. We touched upon but did not formalize the solutions for cases where the manager and/or the species under consideration have vector-valued traits. We note how the manager’s strategy may alter the number of coexisting strategies within the ESS. A manager’s harvest strategy of keeping medium-sized adults while releasing smaller (juvenile or young adults) and larger (high reproductive potential) individuals, such as occurs in lobster fisheries could select for two species of lobster where there had been one—a species that only breeds below the lower threshold and one that waits to breed until above [87]. We did not consider all of the important stability properties associated with convergence stability, NIS and mutual invasibility that might influence the speed and possibility of the species achieving its ESS once subjected to the manager’s strategy. The examples investigated in this paper do not explicitly account for spatial structures or constraints. It remains for future studies to investigate how spatial structures, as for example studied in [50,88], can be included in the SEG framework.

Finally, in terms of the applications discussed here, we did not consider the manager’s utility derived from the transient dynamics nor whether permanently aiming for transients would be the best strategy, such as strategies of applying or withholding a pesticide or cancer therapy when the abundance of the pest is above or below some threshold. SEGs can and should be extended to differential games where time-dependent, optimal control strategies become an option for the manager. An important context for extending SEGs concerns cases where there are multiple stakeholders, each with different objectives and perhaps access to different strategies. The SEG now includes a game among the stakeholders in addition to their intentional and collateral effects on the species ESS. Along this line, there may be a diversity of different species within the community under management. For instance, there is mounting evidence that harvesting fish in coastal fisheries may be releasing octopus (a different G-function) from predation or competition, thus increasing their numbers and inviting the expansion of octopus harvesting [89,90]. SEGs with multiple stakeholders and multiple G-functions among the species of interest may be quite frequent.

In conclusion, 50 years saw EGT grow into a substantial body of models, theories and applications that are now central to studies of evolutionary ecology. In bioeconomic games of managing evolving resources and pests, we see the emergence of SEG theory. And while some of us may not be alive to see what happens in the next 50 years, we believe that SEG theory will be an essential body of mathematics vital for managing humanity's relationship with nature.

## Data Availability

Analysis code is available online at https://github.com/alexsteininfo/SEGTheory. The data are provided in electronic supplementary material [91].
